# The short-term impact of the 2020 pandemic lockdown on employment in Greece

**DOI:** 10.1007/s00181-023-02381-2

**Published:** 2023-02-16

**Authors:** Gordon Betcherman, Nicholas Giannakopoulos, Ioannis Laliotis, Ioanna Pantelaiou, Mauro Testaverde, Giannis Tzimas

**Affiliations:** 1 School of International Development and Global Studies, University of Ottawa, IZA, Ottawa, Canada; 2 Department of Economics, University of Patras, Patras, Greece; 3 University of Peloponnese, Peloponnese, Greece; 4 Global Labor Organization, City University of London, London, UK; 5 Athens University of Economics and Business, Athens, Greece; 6 World Bank, Washington, D.C., USA

**Keywords:** Employment, Job hiring, Job separation, COVID-19, Greece, E24, J21, J63, J68

## Abstract

This paper analyzes the short-term employment impact of the COVID-19 lockdown in Greece during the first few months following the pandemic onset. During the initial lockdown period, aggregate employment was lower by almost 9 percentage points than it would have been expected based on pre-pandemic employment trends. However, due to a government intervention that prohibited layoffs, this was not due to higher separation rates. The overall short-term employment impact was due to lower hiring rates. To uncover the mechanism behind this, we use a difference-in-differences framework, and show that tourism-related activities, which are exposed to seasonal variation, had significantly lower employment entry rates in the months following the pandemic onset compared to non-tourism activities. Our results highlight the relevance of the timing of unanticipated shocks in economies with strong seasonal patterns, and the relative effectiveness of policy interventions to partly absorb the consequences of such shocks.

## Introduction

Since the onset of the COVID-19 pandemic, the consequences for economies and labor markets around the world have been immense (e.g., Alstadsæter et al. 2020; Béland et al. 2022; Cowan 2020; Coibion et al. 2020; Cajner et al. 2020; Forsythe et al. 2020; Campello et al. 2020; Bauer and Weber 2021; Kong and Prinz 2020; Goolsbee and Syverson 2020; Aum et al. 2021). As an exogenous, transitory shock, it directly impacted economic activity through a plummeting consumer demand (Kong and Prinz 2020; Kim et al. 2022; Jordà et al. 2022), and it generated heterogeneous effects across several dimensions of economic activity (Barrot et al. 2021; Guerrieri et al. 2022). Also, it forced governments to impose restrictions on both mobility and economic activity, in an attempt to mitigate the virus transmission. However, the demand for goods and services decreased mainly because individuals adjusted their behavior to protect themselves from being infected, especially in times with overwhelmed healthcare systems, and months before the vaccine rollout (Goolsbee and Syverson 2020; Kapetanios et al. 2022). In the short-run, such behavioral adjustments were responsible for the worsening economic conditions (Baek et al. 2021). As a result, the demand for labor decreased. At the same time, labor supply fell because people stayed at home due to concerns about individual and public health. Overall, the immediate responses in terms of employment came through higher separation rates (Adams-Prassl et al. 2020), fewer hires (Guven et al. 2020; Aum et al. 2021; Casarico and Lattanzio 2022; Zou et al. 2022), and fewer vacancy postings (Hensvik et al. 2021; Bamieh and Ziegler 2020).

Despite the timing of the pandemic onset being nearly common for everyone, the short-run labor market effects depended on the structure of economic activity (i.e., region-specific sectoral variation). Therefore, searching for the mechanisms behind the observed short-term employment adjustments will provide insights regarding the process of structural labor reallocation, compatible with a theoretical framework of labor market frictions (Şahin et al. 2014). As Pizzinelli and Shibata (2022) point out, the COVID-19 pandemic did not set in motion a large process of structural reallocation, since the initial exposure of different sectors to the pandemic resulted primarily from the short-run impact of virus transmission concerns, behavioral adjustments, and various mitigation policies. To provide some answers regarding the above, this paper attempts to identify (a) the short-run impact of the pandemic on labor market outcomes, and (b) whether the observed short-term employment losses were due to lower hiring or higher separation rates. Using individual level data from the Greek Labour Force Survey we rely on the region-specific variation in economic activity, and define sectors that were severely affected by mobility restrictions (e.g. transportation, accommodation, restaurants etc.) versus those that were not (e.g. electricity, public administration, education etc.). We should mention three interesting features about the Greek case. First, the economy is traditionally exposed to strong seasonal patterns (OECD 2020).^1^ Second, before the pandemic, the country was recovering from a prolonged period of economic recession.^2^ Third, the initial policy response after the pandemic onset was focused on protecting existing jobs and providing financial support to firms mostly affected. A key condition for firms to receive support was that they temporarily prohibited layoffs, a decision aligned with the general consensus among EU policy makers at that time (Giupponi et al. 2022; Ando et al. 2022; Schelkle 2021).^3^ Constructing sector-region cells on a quarterly basis, and under a difference-in-differences (DiD) framework we compare labor market outcomes (job finding, job separation, employment, and labor force participation rates) before and during the early pandemic phase between affected and not affected cells.

Our estimates show that employment rate was around 9 p.p. lower relative to its pre-pandemic level. Employment losses were due to declines in the demand for labor after the pandemic onset in tourism-exposed cells, which primarily drive the seasonal employment patterns.^4^ This result was solely due to a lower hiring rather than a higher separation rate. Our findings are in line with evidence for the US (Forsythe et al. 2020), Canada (Larue 2021), Korea (Aum et al. 2021), and Sweden (Hensvik et al. 2020), where the employment slowdowns in the early 2020 were due to fewer vacancies in the hospitality sectors. Separation rates were not significantly increased due to the lockdown and this is attributed to the policy measures that protected existing jobs by prohibiting layoffs in suspended sectors. This conclusion contrasts the increased separation rates reported by Adams-Prassl et al. (2020) for the US and Lemieux et al. (2020) for Canada, where job retention was less of a priority. Our findings have important policy implications regarding the severity of labor market disruptions as responses to the nature and the timing of exogenous shocks that are unrelated to the functioning of the economy and the labor market.

The remainder of this paper is organized as follows. Section 2 presents some stylized facts on mobility and labor market trends before and during the early pandemic period in Greece, and it briefly describes the lockdown and mitigation measures introduced by the government. Section 3 presents the identification strategy, the data sources and provides summary statistics. Section 4 outlines the empirical methodology and presents the estimation results. Section 5 concludes.

## The COVID-19 outbreak in Greece

The first COVID-19 case was confirmed on February 26, 2020. The outbreak peaked in early April with about 100 new cases per day. This relatively low number of cases was mostly due to the fact that the government reacted quickly, within three weeks after the initial recorded infection, and adopted policy measures that restrained the initial spread, including school closings, closing of all non-essential workplaces, and finally, issuing a general stay-at-home order.^5^ Appendix Fig. 6 plots the timeline of the pandemic in terms of infections and public policy responses. Early in May, the government lifted the stay-at-home order, followed by the opening of schools, and a gradual reopening of commercial and workplace activities by the end of May. Similar to other countries, mobility patterns changed dramatically during the first pandemic wave (Appendix Fig. 7).^6^ Public transit and visits to workplaces and non-essential shops declined by 50%-80% compared to their pre-pandemic levels. At the same time, visits to essential retail stores (grocery stores and pharmacies) were affected to a clearly lower extent. There was a partial return towards the pre-pandemic baseline after the first lockdown was lifted. According to the Hellenic Statistical Authority (ELSTAT) April 2020 press release, suspended sectors covered 14.6% of firms and 25.4% of employees. Accommodation was the most affected sector (87% of firms and 94.2% of workers) and the most exposed regions were South Aegean and the Ionian Islands, where the share of suspended firms was 34.4% and 29.8%, respectively. The severity of the pandemic on tourism-exposed sectors was also reflected in a 94% drop in total revenues in the accommodation sector during the second quarter of 2020, when the overall decline in the economy was 25% (ELSTAT). These suspensions and mobility restrictions concurred with the seasonal peak in job hiring. According to pre-pandemic Ministry of Labor and Social Affairs monthly press releases, new hires during the second quarter of 2019 accounted for 30% of all hires during the year, and half of those were made by firms in the accommodation sector. This sector is very important for the Greek labor market. In 2019, it accounted for 43% of the total number of hires nationally, with the majority of these registered during the second and third quarter of the year.

**Fig. 1:**
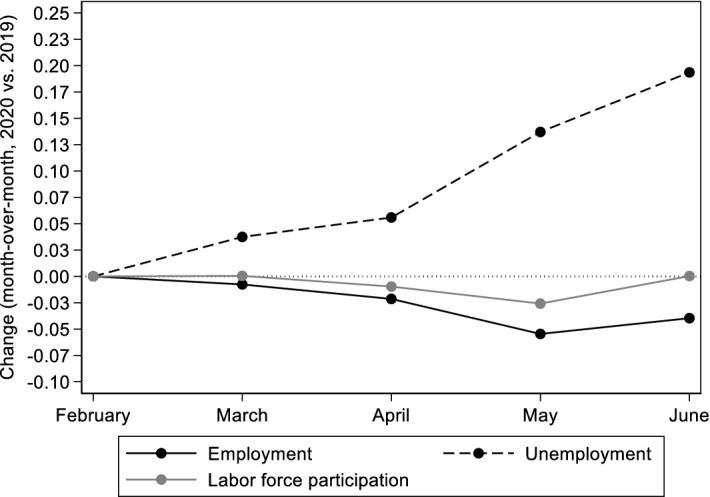
Month-over-month change in the within-year evolution of aggregate labor market indicators. Source: Hellenic Statistical Authority (ELSTAT) Labor Force Survey (LFS) Monthly Estimates (seasonally adjusted, persons 15–74 years old). Notes: Each dot represents the difference between (a) the % change in labor market outcomes of each month in 2019 relative to February of that year, and (b) the % change in labor market outcomes of each month in 2020 relative to February of that year

The demand shock and the subsequent restrictions imposed by the government resulted in a major economic slowdown with significant consequences for firms and workers (Economides et al. 2022). In 2020, real GDP declined by 8.2%, compared to 2019 (ELSTAT).^7^ Seasonally adjusted ELSTAT estimates showed that in May 2020 (relative to February 2020) the labor force participation rate and the employment rate decreased by 0.9 and 1.6 p.p., respectively, while the unemployment rate increased by 1.5 p.p. Figure 1 shows the year over year (2020 vs. 2019) change for each month (in each year February is used as the reference month) of basic aggregate labor market indicators (employment, unemployment, and labor force participation rates).^8^ These changes illustrate how employment and non-employment rates evolved during the early months of the pandemic. Assuming that the trend changes in 2020 reflect the impact of COVID-19, we observe that employment and labor force participation rates were lower, while the unemployment rate was higher in May 2020, compared to 2019.

To keep the economy afloat, the government mobilized a support package amounting to €6.8 billion (3.5% of GDP) in March and April, and approved an additional package of €24 billion in May in order to stimulate the restart of the economy. The first legislative act to support employers (March 11) included various firm-level liquidity measures.^9^ On March 18, the Ministry of Finance and the Ministry of Labor and Social Affairs introduced new measures,^10^ including an €800 stipend (covering the period from March 15 to April 30, eventually extended through May) to workers whose contracts had been suspended because of the suspension of operations of their workplaces. By early May, approximately 1.2 million employees and 550,000 self-employed and freelancers benefited from this support scheme.^11^ Unemployment and long-term unemployment benefits, and unemployment allowance payments for the self-employed were extended by two months for those whose entitlement ended on March 31, and it was further extended to cover those whose entitlements ended at the end of April and May.^12^ In addition, a lump sum stipend of €400 was introduced for 155,000 long-term unemployed individuals, registered with the Public Employment Agency (OAED) from April 1, 2019 who were maintaining their status until April 16, 2020 and were not receiving any other state benefit.^13^ In terms of numbers affected and financial commitment, the government’s mitigation measures emphasized the preservation of employment in firms operating in suspended sectors. To receive the benefits, a key condition was that affected firms were obliged not to reduce their pre-pandemic headcount. In fact, layoffs in suspended sectors of economic activity were prohibited from March 18 until June 16 when the restriction was lifted. Estimates from the National Institute of Labor and Human Resources in Greece (EIEAD 2021) show that during March–April 2020, almost half of the total salaried employment in Greece was affected by those schemes.

Aggregate evidence on whether the employment changes were due to changes in job hiring and/or job separation rates can be derived by daily data on job flows. Results presented in the Appendix sub-section A.1 show that the employment decline, by 7.8 p.p., (column 10 of Table 5) was entirely due to the collapse in job hiring rather than increases in job separations. For instance, the post-COVID hiring rate was 23.1 p.p. lower compared to what was expected, and almost half of this decline was due to the decrease in full-time hires. Similarly, the separation rate during the first pandemic wave was 14 p.p. lower and the majority of this decline was due to the 6.9 p.p. reduction in the contract termination rate. Moreover, to complement the evidence on how the labor market responded after the pandemic onset, we used daily vacancy data. The results (Appendix sub-section A.2) indicate a sharp decline in job vacancies due to the implementation of workplace restrictions in mid-March (Fig. 9), particularly in the accommodation and food services sectors (Table 6).

## Identification strategy and data sources

During normal times, firms base their employment decisions on their experiences and expectations, mostly shaped from seasonal patterns, regarding the demand for their goods and services. The pandemic outbreak interrupted this normality. The Greek government imposed horizontal mobility restrictions in all regions (international travel restrictions were also imposed), eliminating the ability of people to move to places outside their region of residence. However, despite the horizontal implementation of those restrictions, their impact on economic activity should not be expected to be geographically homogenous. Instead, it should depend on the exposure of each region to activities related to mobility of people across regions. Therefore, productive units whose economic activity depends strongly on interregional mobility should be more adversely affected, compared to productive units less economically dependent on such mobility. For example, people who planned to visit specific locations and spend some nights in local hotels could not do so in the presence of mobility restrictions. Hence, revenues in the associated sectors could not be realized. Therefore, employment in firms with tourism-related activities should be more affected compared to employment in firms not dependent on such activities.

**Fig. 2:**
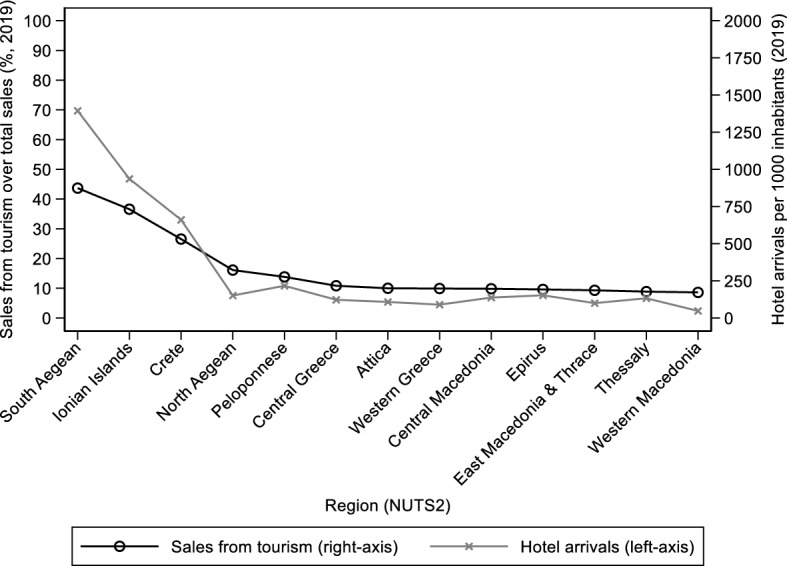
Share of sales from tourism-related activities over total sales and arrivals in tourist accommodation establishments per 1,000 inhabitants by region (NUTS-2). Source: Hellenic Statistical Authority (ELSTAT), Statistical Business Register 2019 (https://www.statistics.gr/en/statistics/-/publication/SBR01/-), Arrivals in hotels and similar establishments by region 2019 () and Estimated Population 2019 (). Notes: Each point on the line measured on the right-axis is the region-specific share (%) of total sales generated by tourism-related sectors and each point on the line measured on the left-axis is the region-specific ratio of arrivals in hotels and similar establishments over 1,000 inhabitants

To empirically identify the short-run labor market impact of the pandemic, we exploit the variation of the economic activity (2-digit NACE Rev. 2 classification, 88 sectors) within each region (NUTS-2 classification, 13 regions). We classify sectors into two groups, a treated (Transportation, Accommodation, Food & beverages, Real estate, Travel agencies, Creative activities, Gambling & Betting, and Sports activities) and an untreated one, based on the International Recommendations for Tourism Statistics 2008, UN/UNWTO taxonomy (Appendix, Table 7). To explore whether the above grouping identifies differences across regions regarding their exposure on those activities that are mostly affected by mobility restrictions, we use pre-pandemic (2019) information from ELSTAT on sales (turnover), and calculate for each region the share of sales generated by the treated sectors (as a percentage of that region’s total sales). In addition, we calculate the number of total arrivals (natives plus foreigners) in tourist accommodation establishments per 1,000 inhabitants (using the regional population of 2019).^14^ After sorting regions based on their tourism-related share of total sales, there are four regions that are relatively more exposed to tourism-related activities (South Aegean, Ionian Islands, Crete and North Aegean) compared to other regions (Fig. 2). Moreover, those tourism-exposed regions have the highest number of arrivals in tourist accommodation establishments per 1,000 inhabitants. Thus, grouping sectors into treated and untreated ones will help us to identify the short-run labor market impact of the pandemic, given that the sectoral composition is not homogenous across regions.

**Fig. 3:**
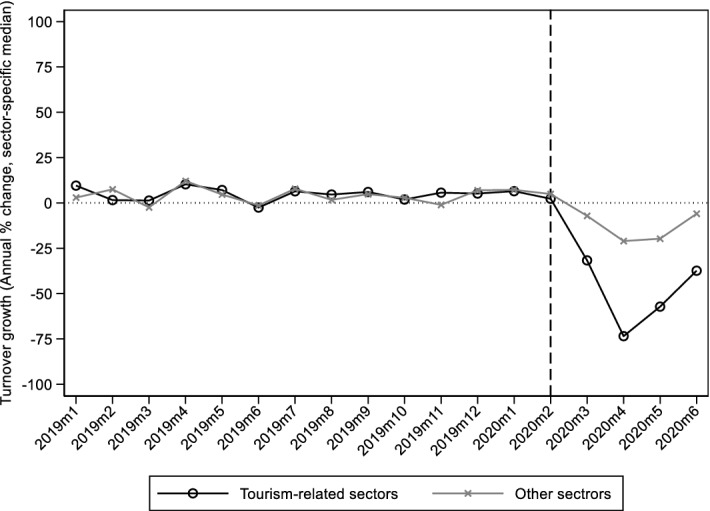
Turnover growth in tourism related sectors and in other sectors. Source: Hellenic Statistical Authority (ELSTAT), Turnover from Administrative Sources (2018M1-20202M6). https://www.statistics.gr/en/statistics/-/publication/SBR02/- Notes: Each point on the graph shows the median turnover annual growth (% change), at the national level, for sectors in tourism-related activities (treated) and for other sectors (untreated).The grouping of sectors (NACE Rev 2, 2 digit) in tourism-related and other sectors is shown in Table 7 (Appendix)

**Fig. 4:**
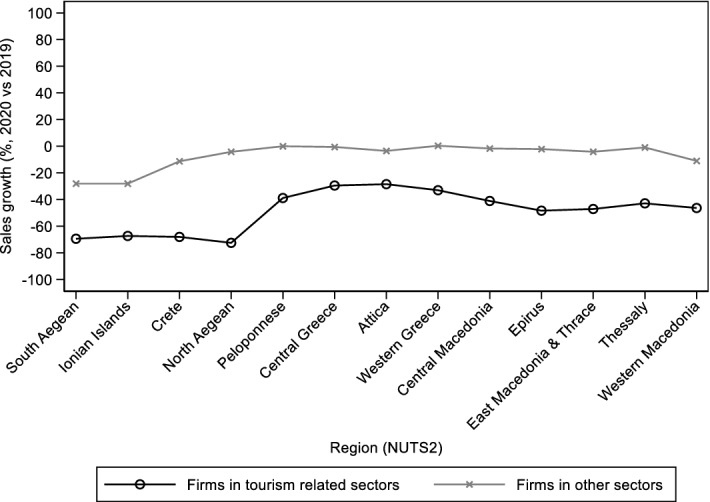
Sales growth across regions for firms in tourism related sectors and firms in other sectors. Source: ICAP Data.Prisma (2019 and 2020). https://www.icapdataprisma.com/ Notes: Each point on the graph shows the region-specific median sales growth for firms in tourism-related sectors (treated) and firms in other sectors (untreated). The grouping of firms in the above two categories is based on their sectoral affiliation (NACE Rev 2, 2 digit) as reported in the ICAP Data.Prisma dataset and the classification of tourism-related activities shown in Table 7 (Appendix). There are 42,767 firms with non-missing information on sales in 2019 and 2020. For each firm, we calculated the percentage change in total sales between 2019 and 2020 and then we got for each region the median sales growth for the treated and untreated groups

To validate the ability of our treatment assignment strategy to capture the responsiveness of economic activity on mobility restrictions, we show the monthly evolution of annual sales growth between January 2018 and June 2020 for treated and untreated sectors, at the national level (Fig. 3).^15^ Sales dropped considerably more in treated relative to the untreated sectors, e.g. 75% versus 25% in April 2020, respectively. This finding was also confirmed using firm-level information from the ICAP Data.Prisma dataset, that provides balance sheet data and information about the sector of economic activity (2-digit NACE Rev. 2) and NUTS-2 region for each firm. Using those data, we calculated the annual sales growth for each firm between 2020 and 2019. Then, for each region, we calculated the median sales growth for firms operating in tourism-related (treated) and untreated sectors (Fig. 4). We observe that the annual sales growth in the treated sectors was lower in regions that are more exposed to tourism-related activities.

Based on the above strategy to identify treated and control units, we calculated a series of labor market outcomes for each sector-region cell using individual-level data from the Labor Force Survey (LFS) covering the period 2015Q1-2020Q2. The LFS contains information on demographic, human capital, employment status and economic characteristics of surveyed households and their members. In particular, to construct the labor market outcomes of interest, we use information on the region of residence, and on current and past sector of economic activity at the individual level. More specifically, for individuals who are (a) employed in the surveyed quarter we know whether they started working during that quarter and (b) non-employed in the surveyed quarter we know whether they stopped working during that quarter. Although information for the non-employed who left or lost their job in the last two years is available, we only considered those who left or lost their job in the previous year, in order to be more accurate about their sectoral affiliation. Therefore, we have a pool of individuals who became employed during a surveyed quarter in a specific sector and region or continue to be non-employed over the last 4 quarters (but for which the sector of economic activity of the last employment and current region of residence is known). The constructed labour market outcomes of interest are quarterly series on job finding, job separation, employment, and labor force participation rates for each sector-region cell (1,144 cells in total). For the job finding rate, the numerator is the number of individuals who started working during the current quarter and the denominator is the number of individuals who were not employed over the last 4 quarters. For the job separation rate, we first defined a pool of individuals who either became non-employed during the current quarter in a specific sector and region or continued to be employed; this is the denominator. The numerator is the number of individuals who stopped working in the current quarter and the denominator is the number of employed individuals in that quarter. For the employment rate, the number of working individuals in the current quarter was divided by the sum of the currently working individuals plus the number of non-employed who stopped working the last 4 quarters. For the labor force participation rate, the sum of those working in the current quarter plus those currently unemployed (i.e. those who lost their job during the last 4 quarters) was divided by the sum of those currently working individuals plus the unemployed and inactive individuals who lost their job during the last 4 quarters. All series are weighted by the LFS population weights.

**Fig. 5:**
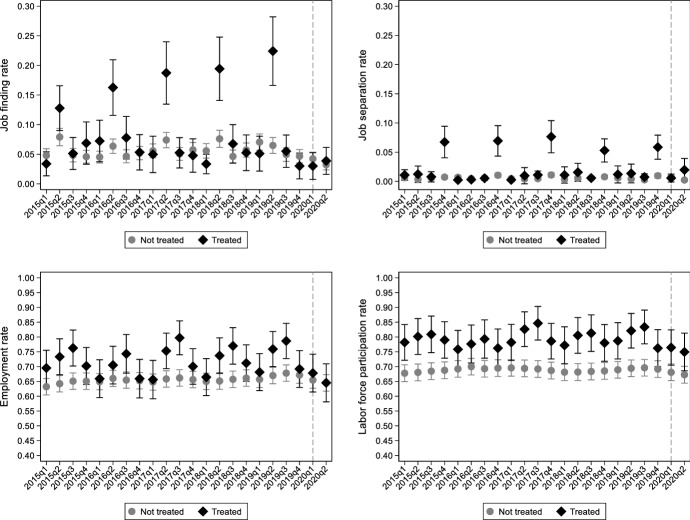
Labor market outcomes before and during the pandemic in treated and untreated cells. Source: Hellenic Statistical Authority (ELSTAT), Labor Force Survey (LFS), Public Use Microdata files (2015Q1-2020Q2). Notes: OLS estimates. Outcomes are the average rates in each quarter and group (treated and not treated sector-region cells). The average job finding rate is the share of individuals who started working in the current quarter over the number of individuals who are non-employed during the last four quarters. The average job separation rate is the share of individuals who stopped working in the current quarter over the number of currently employed individuals. The average employment rate is the share of currently employed individuals over the number of individuals who are non-employed during the last four quarters. The average labor force participation rate is the share of currently employed or unemployed individuals over the number of individuals who are either employed in current quarter or are non-employed during the last four quarters. All rates are weighted using the ELSTAT population weights (individuals in the age group 15–64). Dashed vertical lines correspond to the pandemic onset (2020Q1)

Figure 5 displays how labor market outcomes trended for treated and untreated cells before and during the early pandemic period. Regarding the job finding rate, our treatment assignment fully captures the seasonal character of the Greek labor market according to which new hires in treated cells peak during the second quarter each year before the pandemic (2015–2019). For untreated cells, however, within year fluctuations were limited. In 2020Q2, the job finding rate for treated cells did not increase as in previous years, and it remained at the levels of precedent pre-pandemic quarters. Seasonality is also evident in the case of job separation. Treated and untreated cells trended similarly before the pandemic onset. However, separation rates in treated and untreated cells were not different after the pandemic onset. Regarding the employment rate, we observe strong seasonal patterns in the pre-pandemic year for the treated cells while this is not the case for the untreated ones. In particular, during the pre-pandemic period, the employment rate, on average, was around 70% and it increased notably during the second and third quarters of the year, while it decreased in the first and fourth quarters. Combing the evidence for the job finding and job separation rates, it seems that in tourism-related cells, within-year fluctuations in hires drove the seasonal employment patterns more relative to fluctuations in job separations. In the untreated cells, the employment rate did not fluctuate within the year during the pre-pandemic period. It should be noted that employment rates for both treated and untreated cells were trending slightly upwards since 2015, reflecting the mild recovery in the Greek economy in the years after the 2008–2009 financial crisis. However, in 2020Q2, the employment rate for tourism-related cells reversed its pre-pandemic trend and dropped to 60%, which is equal in magnitude to that of untreated cells. Moreover, in 2020Q2, employment rate in untreated cells did not considerably change from its pre-pandemic trend. Regarding labor force participation rate in treated cells (2020Q1-2020Q2), it decreased both relative to its pre-pandemic trend, and relative to how it evolved in untreated cells.

Mean differences in the labor market outcomes by treatment group and period are shown in Table 1. Given the pandemic onset in February and the quarterly frequency of the LFS data, the first two quarters of 2019 are the pre-pandemic period, and the first two quarters of 2020 are the pandemic period. Regarding the second quarter each year, the job finding rate for the treated (untreated) cells was 22.4% (6.5%) in 2019 and it dropped to 3.9% (3.2%) in 2020. These within-group differences correspond to a 3.3 p.p. reduction for the untreated cells (column 3) and 18.5 p.p. reduction for the treated ones (column 6). The between-groups difference in the above two within-group differences (column 7) is a 15.3 p.p. reduction for the treated cells over and above the developments that took place over time in the untreated cells. The job separation rates did not change significantly for either group although job separation for treated cells was higher in both periods. Employment rate for the untreated did not change significantly before and during the first pandemic wave, fluctuating around 65%-67%. However, for the treated cells, this figure dropped from 75.9% in 2019 to 64.5% in 2020. The between-groups difference in the above two within-group differences (column 7) results in a reduction of 8.9 p.p. in the treated units over and above the developments that took place across time in the untreated units. The labor force participation rate decreased for both groups during the first pandemic wave but mostly for the tourism-related cells; the net change in column 7 represents a reduction of 5 p.p. Repeating the same exercise for the first quarters of 2019 and 2020 does not reveal any significant differences between treated and untreated cells in the two periods. However, the results regarding the first quarters should be viewed with caution because 2020Q1 includes a few weeks after the pandemic onset.

**Table 1:** Mean differences in labor market outcomes by treatment group and period

	Not treated			Treated			
	2019 [1]	2020 [2]	Within difference = [2]-[1] [3]	2019 [4]	2020[5]	Within difference = [5]-[4] [6]	Overall difference = [6]-[3][7]
*Second quarter*							
Job finding	0.065	0.032	– 0.033*** (0.008)	0.224	0.039	– 0.185*** (0.032)	– 0.153*** (0.031)
Job separation	0.004	0.002	– 0.002 (0.002)	0.013	0.019	0.006 (0.013)	0.008 (0.013)
Employment	0.669	0.644	– 0.025 (0.020)	0.759	0.645	– 0.114** (0.045)	– 0.089*** (0.034)
Labor force participation	0.694	0.672	– 0.022 (0.020)	0.821	0.749	– 0.072 (0.044)	– 0.050 (0.031)
*First quarter*							
Job finding	0.070	0.042	– 0.028*** (0.009)	0.051	0.030	– 0.021 (0.019)	0.007 (0.025)
Job separation	0.006	0.007	0.001 (0.002)	0.012	0.006	– 0.006 (0.008)	– 0.008 (0.008)
Employment	0.656	0.654	– 0.002 (0.020)	0.681	0.678	– 0.003 (0.045)	– 0.001 (0.056)
Labor force participation	0.689	0.680	– 0.008 (0.020)	0.787	0.764	– 0.023 (0.044)	– 0.014 (0.056)

Source: Hellenic Statistical Authority (ELSTAT), Labor Force Survey (LFS) Public Use Microdata files

Notes: All averages are population weighted. The average job finding rate is the share of individuals who started working in the current quarter over the number of individuals who are non-employed during the last four quarters. The average job separation rate is the share of individuals who stopped working in the current quarter over the number of individuals who are currently employed. The average employment rate is the share of currently employed individuals over the number of individuals who are non-employed during the last four quarters. The average labor force participation rate is the share of individuals who are currently employed or unemployed over the number of individuals who are either employed in current quarter or are non-employed the last four quarters. Standard errors in parentheses. Asterisks ***, ** and * denote statistical significance at the 1%, 5% and 10% level, respectively

We also calculated averages for the composition of each sector-region cell in 2019Q2 (pre-pandemic period) regarding age, gender, country of birth, marital status, education, and occupation. Table 2 presents the pre-pandemic composition of the LFS sample, in terms of demographic, human capital, occupation, sector, and location characteristics. In the treated group, 70% of respondents were affiliated to the accommodation and food services sectors. Moreover, respondents in treated cells were slightly younger, not married, less educated, employed in sales and clerical jobs, and located in tourism-dependent regions.

**Table 2:** Sample composition (total and by treatment group)

Individual characteristics	All [1]	Not treated [2]	Treated [3]
Age (in years)	44.50	45.10	41.30
Gender	0.436	0.435	0.438
Foreign-born	0.082	0.070	0.142
Married	0.668	0.684	0.589
Primary education	0.133	0.135	0.124
Secondary education	0.552	0.519	0.713
Tertiary education	0.315	0.346	0.164
Occupation (ISCO-08, 1 digit)			
Managers	0.026	0.022	0.045
Professionals	0.164	0.193	0.023
Technicians	0.083	0.090	0.051
Clerks	0.105	0.103	0.119
Sales workers	0.236	0.194	0.445
Agricultural workers	0.139	0.168	0.001
Crafts	0.094	0.110	0.018
Assemblers	0.066	0.049	0.148
Elementary	0.086	0.073	0.151
Sector (NACE Rev 2. 1 digit)			
Agriculture, forestry and fishing	0.154	0.185	0.000
Mining and quarrying	0.004	0.005	0.000
Manufacturing	0.090	0.109	0.000
Electricity, gas, steam etc	0.008	0.010	0.000
Water supply; sewerage, etc	0.008	0.010	0.000
Construction	0.040	0.048	0.000
Wholesale and retail trade	0.164	0.198	0.000
Transportation and storage	0.047	0.019	0.182
Accommodation and food service etc	0.120	0.000	0.705
Information and communication	0.019	0.022	0.000
Financial and insurance activities	0.016	0.019	0.000
Real estate activities	0.001	0.000	0.008
Professional, scientific and technical	0.045	0.054	0.000
Administrative and support service etc	0.023	0.018	0.044
Public administration etc	0.087	0.105	0.000
Education	0.080	0.097	0.000
Human health and social work activities	0.056	0.067	0.000
Arts, entertainment and recreation	0.012	0.002	0.061
Other service activities	0.019	0.022	0.000
Activities of households as employers;	0.006	0.007	0.000
Activities of extraterritorial etc	0.001	0.001	0.000
Region (NUTS-2)			
East Macedonia & Thrace	0.095	0.101	0.066
Central Macedonia	0.145	0.153	0.108
Western Macedonia	0.032	0.035	0.018
Thessaly	0.046	0.047	0.044
Epirus	0.060	0.063	0.041
Ionian Islands	0.037	0.025	0.095
Western Greece	0.063	0.065	0.053
Central Greece	0.055	0.059	0.038
Attica	0.228	0.234	0.201
Peloponnese	0.072	0.074	0.063
North Aegean	0.030	0.029	0.035
South Aegean	0.052	0.037	0.122
Crete	0.085	0.078	0.116
Observations (persons)	19,728	16,337	3,351

Source: Hellenic Statistical Authority (ELSTAT), Labor Force Survey (LFS)

Notes: All means are weighted by the LFS survey weights. Data are from the 2019Q2 wave

## Empirical modelling and results

Our primary units of analysis are sectors in each region observed in quarters before and after the pandemic onset. As described in Sect. 3, sectors were classified into treated and untreated ones based on the grouping reported in Table 7 (Appendix), and sectoral composition varies by region. Hence, we rely on a DiD framework to compare labor market outcomes between sector-region cells that should be more affected, relative to those less affected, by the economic slowdown due to the lockdown. We expect that employment-related outcomes in treated cells should have exhibited more substantial adjustments compared to untreated cells. Furthermore, we also constructed an alternative treatment definition based on the economic activity that was suspended by governmental orders (also shown in Table A.3). This should provide some evidence on whether labor market responses differed between suspended and not-suspended cells. For our DiD estimates to be valid, two assumptions should be satisfied, i.e. treatment exogeneity and parallel trends. Given that the mobility restrictions across regions that imposed by the government were homogeneously implemented throughout the country, i.e. without taking into account the structure of the local economy, our treatment indicator is considered to be exogenous (discussed also in Sect. 3). Regarding the parallel trends assumption, Fig. 5 reassures that it is satisfied. Labor market outcomes for treated and untreated cells trended similarly over time before the pandemic onset. Based on the above, our main hypothesis is that following the initial virus spread, any mitigation measures to protect public health should have had more adverse short-run labour market impacts in treated sector-region cells. Due the strongly seasonal character of the Greek labor market, our estimation sample uses data from the second quarters of 2019 and 2020. Our linear DiD regression model with cell-specific fixed effects is the following:

1 $${\Upsilon}_{srt}=\alpha +\beta {P}_{t}+\gamma {T}_{sr}+\delta \left({{P}_{t}\times T}_{sr}\right)+ {u}_{srt}$$

where, ${\Upsilon}_{srt}$ is the labor market outcome of interest for each sector $s$ – region $r$ pair at time $t$, ${P}_{t}$ takes the value of 1 for 2020 and 0 for the pre- pandemic year (2019), ${T}_{sr}$ is a dummy indicator denoting whether a specific sector $s$ in each region $r$, is considered as treated, i.e. it is a tourism-related sector, as defined in Appendix Table 7, $\left({{P}_{t}\times T}_{sr}\right)$ is an interaction term equal to 1 for the treated group in the second quarter of 2020, and ${u}_{srt}$ is the unobserved error term. The parameter of interest $\delta$ indicates the short-term labor market effects of the pandemic on the treated cells. To test whether our DiD results are driven by the suspensions imposed by the government in specific cells after the pandemic onset, we augment Eq. (1) by an interaction term between a dummy indicator that denotes whether economic activity within a cell was suspended (as defined in Table 7. in the Appendix) and the post-pandemic dummy indicator ${P}_{t}$. Furthermore, we estimate Eq. (1) for various sub-samples based on characteristics such as age, gender, country of birth, marital status, and education to examine how the short-term labour market impact of the pandemic varied across groups.

**Table 3:** The impact of COVID-19 pandemic on labor market outcomes: Difference-in-difference (DiD) baseline estimates

	Job finding rate		Job separation rate		Employment rate		Labor force participation rate	
	[1]	[2]	[3]	[4]	[5]	[6]	[7]	[8]
Treated × Post	– .153*** (.031)	– .152*** (.033)	.008 (.013)	.010 (.015)	– .089*** (.034)	– .078** (.034)	– .049 (.032)	– .036 (.032)
Suspended × Post	–	.011 (.011)	–	– .001 (.002)	–	– .028 (.026)	–	– .033 (.025)
Post	– .033*** (.008)	– .033*** (.008)	– .002 (.012)	– .007 (.008)	– .024** (.011)	– .021* (.012)	– .021 (.011)	– .017 (.012)
R-squared (within)	.071	.071	.002	.003	.016	.017	.008	.009
R-squared (overall)	.574	.574	.497	.498	.824	.824	.837	.837
Sectors	88	88	88	88	88	88	88	88
Regions	13	13	13	13	13	13	13	13
Quarters	2	2	2	2	2	2	2	2
Sectors × Regions	1144	1144	1144	1144	1144	1144	1144	1144
Observations	2288	2288	2288	2288	2288	2288	2288	2288

Source: Hellenic Statistical Authority (ELSTAT), Labor Force Survey (LFS)

Notes: OLS estimates. Sample covers the second quarters of 2019 and 2020. The average job finding rate is the share of individuals who currently started working over the number of non-employed individuals in the last two years. The average job separation rate is the share of individuals who currently stopped working over the number of currently employed individuals. The average employment rate is the share of currently employed individuals over the number of non-employed individuals in the last two years. The average labor force participation rate is the share of individuals who are currently employed or unemployed over the number of individuals who are either employed in current quarter or are non-employed during the last two years. All rates are weighted using the ELSTAT population (15–64 years old) weights. The *Post* indicator is equal to 1 for the second quarter of 2020, and 0 otherwise. Standard errors in parentheses are corrected for clustering at the sector-region cell level. Asterisks ***, ** and * denote statistical significance at the 1%, 5% and 10% level, respectively

Table 3 presents our baseline results based on Eq. (1). Job finding rate was 15.3 p.p. lower in treated sector-region cells during the second quarter of 2020, compared to the untreated ones (column 1). Column 2 adds an interaction term between the pandemic period and a suspended sector binary indicator. This was done to examine whether the observed decline was due to the pandemic or the subsequent governmental order to suspend economic activity in certain sectors in order to prevent the virus spread. The results remain unchanged implying that the negative impact was solely driven by the decline in activity in treated sector-region cells rather than the lockdown per se. Job separation rate (columns 3–4) was not statistically different between the treatment and control group during the pandemic. We attribute this finding to the early government intervention that prohibited layoffs in suspended sectors during the early pandemic phase.

However, despite the fact that separations did not increase, the observed decline in finding rates translated into lower employment. The employment rate was 8.9 p.p. lower (column 5) for the treatment group during the early pandemic. Including the interaction term between the pandemic period and the suspended sectors, the decline in the employment rate of the treatment group in the second quarter of 2020 was 7.8 p.p. lower compared to the employment rate of the untreated (column 6). Regarding the labor force participation rate (columns 7–8), the estimated DiD parameter is negative, but not statistically different from zero. It should be noted that a negative estimated coefficient of the post dummy indicator is also reported in all cases but is statistically significant only in the job finding and employment equations, around 3.3 p.p. and 2 p.p., respectively. These baseline results indicate that the COVID-19 pandemic and lockdown did have a negative impact on labor market outcomes in sectors and regions that largely depend on tourism-related activities.^16^ Although the government suspended layoffs in certain sectors that were critical for controlling the spread of within the community, the overall impact on employment rates was driven by a drop in employment entry in treated sector-region cells. Changes in the job separation rate during the pandemic did not seem to determine the observed drop in the employment rate. Lastly, job seeking activity, as captured by the job finding, employment, and labor force participation rates, slowed down in both treated and untreated cells.

Table 4 presents the DiD estimates regarding the heterogeneous impact of the COVID-19 pandemic on labor market outcomes across groups of individuals. For all demographic sub-groups considered here, job finding rates were significantly lower for the treated group during the early pandemic. However, those who were younger, males, native-born, singles, and better educated were the most severely affected groups. It is interesting to notice that the estimated negative impact on job finding rates steadily declines with age. This likely reflects the fact that new hires in tourism-related sectors in the pre-pandemic period were mostly younger individuals, consistent with evidence shown in Table 2. The impact of the pandemic and lockdown on job separation rate was not statistically different from zero for any demographic sub-group. Regarding reductions in employment rates, these were more severe for males, native born, and those with completed secondary and tertiary education. With respect to age, employment rates were lower for individuals aged 30–44 years old. Labor force participation rates were particularly reduced for males in the treated group, a finding that implies a decline in the job seeking behavior of males during the early months of the pandemic. Moreover, all the reported DiD parameters were robust to the inclusion of an additional interaction term between the early pandemic period and the sectors that were suspended by the government.

**Table 4:** The impact of COVID-19 pandemic on labor market outcomes for various sub-groups: Difference-in-difference (DiD) estimates

Sub-group:	Job finding rate		Job separation rate		Employment rate		Labor force participation rate	
	[1]	[2]	[3]	[4]	[5]	[6]	[7]	[8]
Age 15 – 29 years old	– .130*** (.028)	– .130*** (.029)	.017* (.010)	.014 (.011)	– .031 (.035)	– .037 (.036)	.032 (.031)	.032 (.032)
Age 30 – 44 years old	– .107*** (.026)	– .100*** (.029)	.006 (.010)	.008 (.012)	– .076** (.036)	– .078** (.037)	– .030 (.034)	– .030 (.034)
Age 45 – 64 years old	– .092*** (.025)	– .081*** (.025)	– .003 (.005)	– .002 (.004)	– .059* (.036)	– .033 (.035)	– .023 (.036)	– .004 (.034)
Males	– .133*** (.029)	– .135*** (.031)	– .001 (.008)	– .005 (.009)	– .138*** (.035)	– .122*** (.034)	– .104*** (.033)	– .088*** (.033)
Females	– .091*** (.025)	– .075*** (.026)	.008 (.014)	.017 (.015)	– .081*** (.033)	– .075** (.034)	– .025 (.033)	– .012 (.035)
Native-born	– .152*** (.031)	– .153*** (.033)	.001 (.011)	.002 (.012)	– .092*** (.034)	– .076** (.034)	– .053* (.031)	– .038 (.032)
Foreign-born	– .065*** (.021)	– .045*** (.022)	.008 (.007)	.012 (.009)	– .069** (.030)	– .073** (.032)	– .024 (.031)	– .027 (.032)
Married	– .103*** (.026)	– .098*** (.028)	– .001 (.011)	.001 (.012)	– .058 (.036)	– .048 (.034)	– .027 (.033)	– .019 (.032)
Not married	– .144*** (.030)	– .150*** (.033)	.017** (.008)	.017* (.009)	– .085** (.036)	– .085** (.038)	– .032 (.036)	– .017 (.037)
Completed primary education	– .038* (.023)	– .035 (.024)	.001 (.002)	– .003 (.004)	– .055** (.025)	– .044 (.028)	– .006 (.025)	.001 (.027)
Completed secondary education	– .121*** (.027)	– .115*** (.028)	.001 (.012)	.003 (.013)	– .090*** (.033)	– .090*** (.032)	– .058* (.031)	– .056* (.029)
Completed tertiary education	– .128*** (.031)	– .125*** (.033)	.009 (.012)	.011 (.012)	– .116*** (.037)	– .128*** (.040)	– .086** (.037)	– .096** (.040)

Source: Hellenic Statistical Authority (ELSTAT), Labor Force Survey (LFS)

Notes: OLS estimates. Sample covers the second quarters of 2019 and 2020. The average job finding rate is the share of individuals who currently started working over the number of non-employed individuals in the last two years. The average job separation rate is the share of individuals who currently stopped working over the number of currently employed individuals. The average employment rate is the share of currently employed individuals over the number of non-employed individuals in the last two years. The average labor force participation rate is the share of individuals who are currently employed or unemployed over the number of individuals who are either employed in current quarter or are non-employed during the last two years. All rates are weighted using the ELSTAT population (15–64 years old) weights. The *Post* indicator is equal to 1 for the second quarter of 2020, and 0 otherwise. Standard errors in parentheses are corrected for clustering at the sector-region cell level. Asterisks ***, ** and * denote statistical significance at the 1%, 5% and 10% level, respectively

## Conclusions

The COVID-19 pandemic induced severe labor market disruptions worldwide. Following its initial outbreak, governments implemented a series of policies to support firms and their employees, especially those in precarious labor market environments. However, policies and their outcomes varied strongly, depending on the characteristics of each labor market and country-specific idiosyncrasies, e.g. institutions, fiscal and healthcare system capacity. For instance, job retention was not prioritized in Canada where unemployment rose from 6 to 14% and weekly work hours declined by 32% in the early pandemic (Béland et al. 2022; Lemieux et al. 2020). To buffer labor market impacts, furloughing schemes were introduced in the US and the UK, however, 20% and 17% of workers lost their jobs by early April 2020, respectively (Adams-Prassl et al. 2020). In Germany, the eligibility criteria for short-time work became less stringent and the percentage of workers who lost their jobs was considerably lower, i.e. around 5% (Adams-Prassl et al 2020; Mayhew and Anand 2020).

Our paper adds to the literature considering Greece, a country that focused on protecting existing jobs, following a gradual recovery from a prolonged recession and given that a large part of its economy is exposed to seasonal demand for services that involve tasks that cannot be done remotely. Several sectors of economic activity were suspended to restrain the spread of the virus. However, layoffs were prohibited and firms in those sectors could receive financial support conditional on preserving their pre-pandemic headcount. Another distinctive feature of our case is that the national lockdown was imposed during a period when the seasonal, heavily reliant on tourism-related activities (especially in certain regions) economy would normally be gearing up with increased hiring.

To investigate the short-run labor market impacts of the pandemic, we used Labor Force Survey data (2015–2020). We constructed a sector-region panel with information on several labor market outcomes, and we defined cells that were mostly exposed to tourism-related activities and cells that were not affected by tourism-related activities. Under a difference-in-differences framework, we compared the evolution of labor market outcomes for treated and untreated cells before and after the pandemic onset. Our results show that the employment rate in the tourist-affected group fell by approximately 9 p.p. during the first pandemic wave relative to the unaffected group. This reduction was solely due to reduced job finding rates; separation rates did not increase in treated cells due to the layoff prohibitions implemented by the government. Our findings highlight the relevance of policy-making in determining how labor markets adjust to external shocks. For example, Greece (along with some other European countries) emphasized job-retention measures to mitigate the consequences of the pandemic. This resulted in low job separation rates in the early months. Although unemployment modestly increased, it was not because of layoffs. This stands in contrast to countries like the US, the UK and Canada, where unemployment rose quickly as policies emphasized income support more than job protection.

Our findings have important policy implications regarding the nature of labor market disruptions as responses to purely exogenous shocks. The drop in consumer demand due to generalized concerns about the pandemic affected the labor market, however, in the short-run it did not fuel the pool of unemployed with layoffs as in previous crises, e.g. the 2008–2009 one, due to the adopted job retention schemes. Hence, any adverse employment outcomes should be viewed as temporary only, given that the pandemic onset coincided with the time when hires in specific sectors normally peak. Indeed, the gradual re-opening of the economy showed that it was only after the first quarter of 2021 when vacancies increased back to their pre-pandemic level, and unemployment returned to a declining trend. Therefore, the seasonal pattern was not simply postponed, in the sense that hiring did not catch up later on in 2020.

## Appendix

**Fig. 6:**
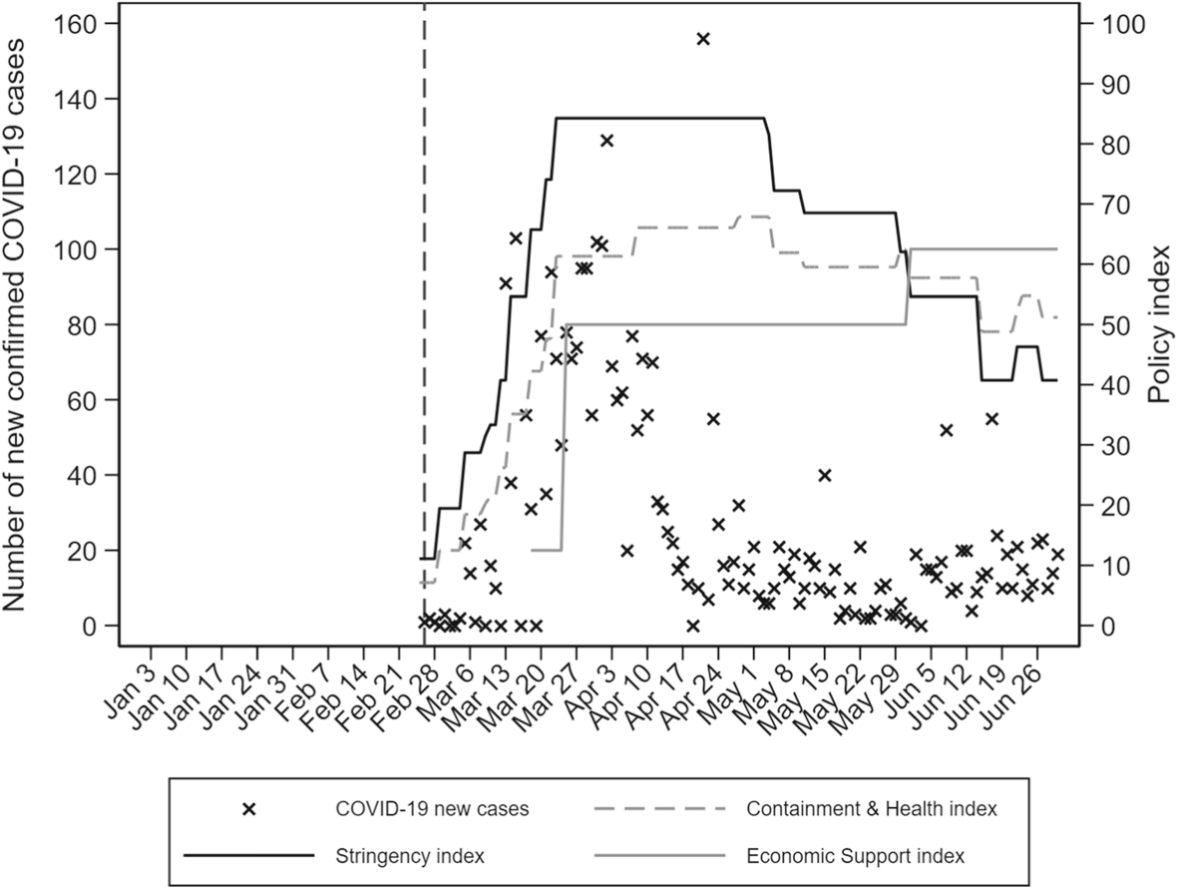
COVID-19 cases and public policy mitigation measures in 2020. Source: Johns Hopkins University; University of Oxford, Blavatnik School of Government; Authors’ calculations. Notes: The Containment and Health index combines lockdown restrictions and closures with measures such as testing policy, contact tracing, short-term healthcare investment in healthcare, and investments in vaccine. The Economic Support index records measures such income support and debt relief. The Stringency index records the strictness of lockdown-style policies that primarily restrict behavior and activities. Vertical dashed line is set in the day when the first COVID-19 case was confirmed (February 26, 2020). The dates on horizontal axis refer to Friday of each week

**Fig. 7:**
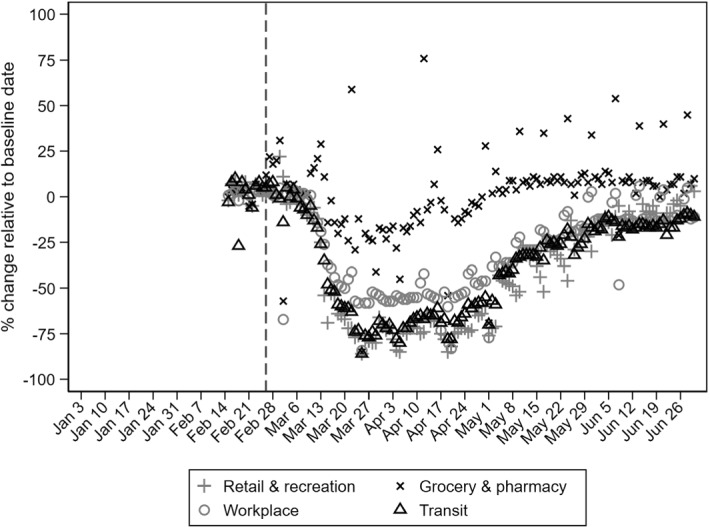
Daily activity for selected indicators in 2020. Source: Google Community Mobility Reports; Authors’ calculations. Notes: Google data are available since February 15^th^, 2020 which is used as the baseline date. Vertical dashed line is set in the day when the first COVID-19 case was confirmed (February 26^th^, 2020). Dates on the horizontal axis refer to Friday of each week

**Fig. 8:**
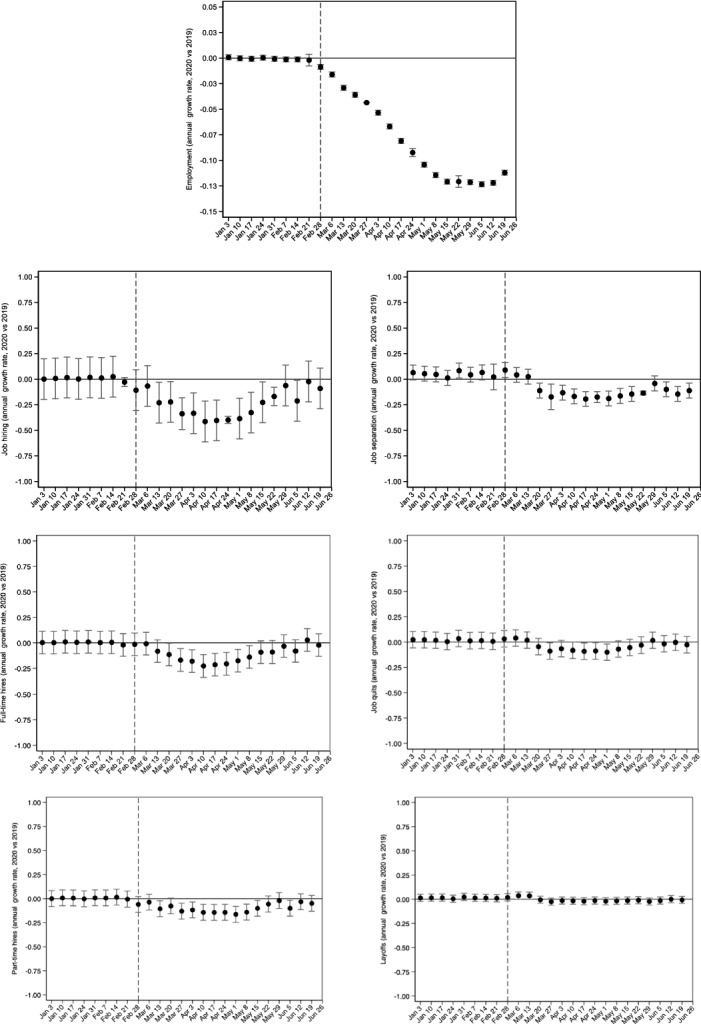

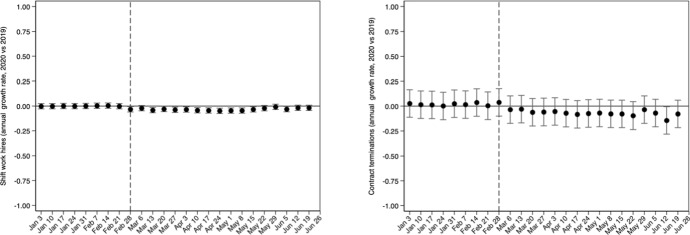
Net annual growth rate of employment, job hiring (total, full-time, part-time, shift work) and job separation (total, quits, layoffs, contract termination) rates. Source: Ministry of Labor and Social Affairs (ERGANI, Monthly Reports); Hellenic Statistical Authority (ELSTAT) Labor Force Survey (LFS) Monthly Estimates (not seasonally adjusted, persons 15–74 years old). Notes: Data cover the period January 01, 2019—June 30, 2020, and refer to the daily new hire and separation rates in salaried jobs at the national level. Daily employment has been constructed by using the 2018 annual stock of salaried employment (ERGANI) as the initial value for January 01, 2019 in which the net job flow is the sum of new hires minus separations. Using the constructed employment stock for January 01, 2019 the same formula (net job flows are added to the daily employment stock) applies to every day in the covered period. Then, the daily employment stock is transformed to a share of monthly population using the ELSTAT monthly population estimate. Job finding and separation are also transformed to shares over the constructed daily stock of employment (average employment of the current and previous day). The vertical axes measure the estimated coefficients (black dots) from fixed effects model specifications, with the constructed rates (employment, job finding and job separation) as dependent variables, and refer to the interaction of the calendar week of the year (in which each day belongs) with a year dummy indicator (2020 vs. 2019). All specifications include day-of-week and month-of-year fixed effects. Vertical capped lines represent 95% confidence intervals based on standard errors that were corrected for heteroskedasticity at the daily level. Vertical dashed lines are set in the day when the first COVID-19 case was identified (February 26^th^, 2020). Dates on horizontal axes refer to the Friday of each week (as in 2020)

**Fig. 9:**
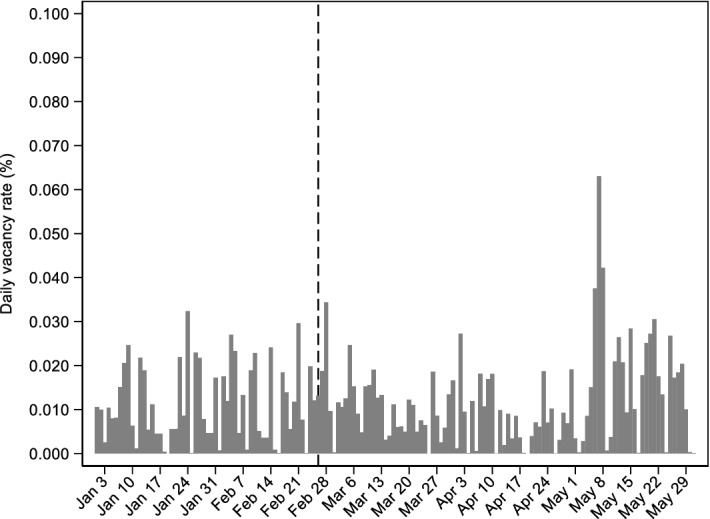
Daily job vacancy rate (2020) based on online job postings. Source: Online job posting platform (www.jobfind.gr); Ministry of Labor and Social Affairs (ERGANI, Monthly Report). Notes: Daily job postings were collected for the period January 01, 2020 – May 31, 2020. The daily vacancy rate was calculated by dividing the number of daily job postings by the 2019 stock of total salaried employment (ERGANI) and the number of daily job postings. Vertical dashed line is set in the day when the first COVID-19 case was identified (February 26, 2020). The dates on horizontal axis refer to the Friday of each week (as in 2020)

**Fig. 10:**
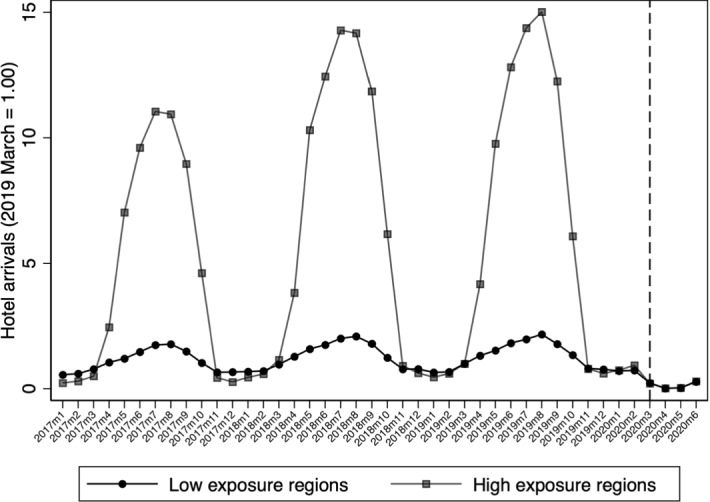
Monthly hotel arrivals index. Source: Hellenic Statistical Authority (ELSTAT), Arrivals in hotels and similar establishments by region (2017M1-2020M6) (https://www.statistics.gr/en/statistics/-/publication/STO12/2020). Notes: Data are from the survey on arrivals and nights spent in hotels, similar establishments and tourist campsites and of short-stay accommodation establishments, conducted by the ELSTAT. We consider total arrivals (natives and foreigners). Low exposure (NUTS-2) regions are: Eastern Macedonia & Thrace, Central Macedonia, Western Macedonia, Epirus, Thessaly, Western Greece, Central Greece, Attica, and Peloponnese. High exposure (NUTS-2) regions are: Ionian Islands, Northern Aegean, Southern Aegean, and Crete. For each group of regions, the index is calculated as the ratio of total monthly arrivals (in hotels and similar establishments) over the number of arrivals in 2019M3

See Figs. 6, 7, 8, 9 and 10 and Tables 5, 6 and 7.

**Table 5:** Job hiring, job separation and employment rates after the pandemic onset: Evidence from daily time series

	Hires				Separations				Employment
	Full-time [1]	Part-time [2]	Shiftwork [3]	Total [4]	Quits [6]	Layoffs [7]	Contract termination [8]	Total [9]	[10]
Treated × Post	– .108*** (.005)	– .093*** (.001)	– .030*** (.003)	– .231*** (.001)	– .051*** (.001)	– .020*** (.001)	– .069*** (.001)	– .140*** (.026)	– .078*** (.001)
Post	.134 (.036)	.078 (.054)	.027 (.019)	.240 (.110)	.022 (.030)	.013 (.010)	.028 (.023)	.065 (.064)	.040 (.038)
R-squared (within)	.567	.730	.773	.683	.655	.555	.417	.531	.939
Day of week	Yes	Yes	Yes	Yes	Yes	Yes	Yes	Yes	Yes
Month dummies	Yes	Yes	Yes	Yes	Yes	Yes	Yes	Yes	Yes
Observations	912	912	912	912	912	912	912	912	912
Not-pandemic days (average 2018–2019)	.124	.114	.036	.274	.114	.049	.114	.277	.239
Pandemic days (average 2018–2019)	.171	.158	.047	.376	.134	.060	.160	.354	.267

Source: Ministry of Labor and Social Affairs (ERGANI, Monthly Reports); Hellenic Statistical Authority (ELSTAT) Labor Force Survey Monthly Estimates (not seasonally adjusted, persons 15–74 years old)

Notes: OLS estimates. Data cover the period between January 01, 2018 and June 30, 2020, and refer to the daily new hires and separations (total and by type) in salaried jobs at the national level. The daily employment series has been constructed by using the 2017 annual national stock of employment in salaried jobs (ERGANI) as the initial value for January 01, 2018 in which the net job flows is the sum of new hires minus separations. Using the constructed employment stock for January 01, 2018 the same formula (net job flows are added up to the daily employment stock) applies to every day in the covered period. Then, the daily employment stock is transformed to a share of monthly population using the ELSTAT monthly population estimate. Job hiring and separation rates (total and by type) are also transformed to shares over the constructed daily stock of employment (average employment of the current and previous day). Therefore, the dependent variables in columns [1]-[9] are the employment shares and the dependent variable in column [10] is the (log) employment to population ratio. The *Treated* indicator refers to the year 2020, and *Post* indicates the period February 27 – June 30 (in all years). All specifications control for day-of-week and month-of-year fixed effects. Heteroskedasticity robust standard errors in parentheses. Asterisks ***, ** and * denote statistical significance at the 1%, 5% and 10% level, respectively

**Table 6:** Shares of occupied jobs and vacancies by sector of economic activity and quarter before and during the early COVID-19 pandemic

NACE Rev. 2 (1-digit)	Occupied jobs (share)						Vacancies (share)					
	Quarter 1			Quarter 2			Quarter 1			Quarter 2		
	2019	2020	Diff	2019	2020	Diff	2019	2020	Diff	2019	2020	Diff
Mining and quarrying	0.00	0.00	0.00	0.00	0.00	0.00	0.00	0.00	0.00	0.00	0.00	0.00
Manufacturing	0.10	0.10	0.00	0.10	0.10	0.00	0.05	0.16	0.11	0.05	0.07	0.02
Electricity, gas, steam and air conditioning supply	0.01	0.01	0.00	0.01	0.01	0.00	0.00	0.00	0.00	0.00	0.01	0.01
Water supply; sewerage, waste management and remediation activities	0.01	0.00	0.00	0.01	0.00	0.00	0.03	0.04	0.01	0.04	0.06	0.02
Construction	0.03	0.02	0.00	0.03	0.02	0.00	0.00	0.00	0.00	0.02	0.02	0.00
Wholesale and retail trade; repair of motor vehicles and motorcycles	0.18	0.19	0.01	0.18	0.20	0.02	0.07	0.10	0.03	0.06	0.11	0.05
Transportation and storage	0.05	0.05	0.01	0.05	0.05	0.01	0.03	0.14	0.11	0.05	0.01	-0.04
Accommodation and food service activities	0.05	0.05	0.00	0.07	0.05	-0.02	0.32	0.08	-0.24	0.33	0.00	-0.33
Information and communication	0.04	0.04	0.00	0.04	0.04	0.00	0.07	0.06	-0.01	0.01	0.04	0.03
Financial and insurance activities	0.03	0.03	0.00	0.03	0.03	0.00	0.01	0.00	0.00	0.00	0.00	0.00
Real estate activities	0.00	0.00	0.00	0.00	0.00	0.00	0.02	0.01	-0.02	0.01	0.00	0.00
Professional, scientific and technical activities	0.02	0.02	0.00	0.02	0.02	0.00	0.07	0.07	0.00	0.05	0.07	0.02
Administrative and support service activities	0.03	0.03	0.00	0.03	0.03	0.00	0.01	0.00	-0.01	0.02	0.06	0.04
Public administration and defence; compulsory social security	0.17	0.16	-0.01	0.16	0.13	-0.03	0.09	0.19	0.10	0.13	0.29	0.17
Education	0.15	0.15	0.00	0.15	0.15	0.01	0.05	0.02	-0.03	0.02	0.04	0.02
Human health and social work activities	0.12	0.12	0.00	0.11	0.13	0.01	0.10	0.10	0.00	0.20	0.16	-0.04
Arts, entertainment and recreation	0.01	0.01	0.00	0.01	0.01	0.00	0.03	0.02	-0.01	0.01	0.01	0.00
Other service activities	0.02	0.02	0.00	0.02	0.02	0.00	0.04	0.01	-0.03	0.01	0.04	0.03
Total	1.00	1.00	-	1.00	1.00	-	1.00	1.00	-	1.00	1.00	-

Source: Eurostat (Greek series: Job vacancy statistics by NACE Rev. 1 activity. Quarterly data, not seasonally adjusted). Job vacancy rate definition is provided in https://ec.europa.eu/eurostat/web/labour-market/job-vacancies

Notes: Diff refers to the difference in shares between 2020 and 2019

**Table 7:** Classification of sectors of economic activity (NACE Rev. 2, 2-digit) as treated (tourism related activities) and suspended sectors (COVID-19 contamination governmental orders)

NACE Rev.2 (2-digit)	Treated	Suspended	NACE Rev.2 (2-digit)	Treated	Suspended
01 Crop and animal production etc	No	No	50 Water transport	Yes	No
02 Forestry and logging	No	No	51 Air transport	Yes	No
03 Fishing and aquaculture	No	No	52 Warehousing for transportation	No	No
05 Mining of coal and lignite	No	No	53 Postal and courier activities	No	No
06 Extraction of crude petroleum etc	No	No	55 Accommodation	Yes	Yes
07 Mining of metal ores	No	No	56 Food and beverage service activities	Yes	No
08 Other mining and quarrying	No	No	58 Publishing activities	No	No
09 Mining support service activities	No	No	59 Motion pictures etc	No	Yes
10 Manufacture of food products	No	No	60 Programming activities etc	No	No
11 Manufacture of beverages	No	No	61 Telecommunications	No	No
12 Manufacture of tobacco products	No	No	62 Computer programming etc	No	No
13 Manufacture of textiles	No	No	63 Information service activities	No	No
14 Manufacture of wearing apparel	No	No	64 Financial service activities etc	No	No
15 Manufacture of leather products	No	No	65 Insurance, reinsurance etc	No	No
16 Manufacture of wood and products etc	No	No	66 Activities to financial services etc	No	No
17 Manufacture of paper products etc	No	No	68 Real estate activities	Yes	No
18 Printing and reproduction of media etc	No	No	69 Legal and accounting activities	No	No
19 Manufacture of coke and products etc	No	No	70 Activities of head offices etc	No	No
20 Manufacture of chemicals products etc	No	No	71 Architectural and engineering etc	No	Yes
21 Manufacture of basic pharmaceutical etc	No	No	72 Scientific research and development	No	No
22 Manufacture of rubber products etc	No	No	73 Advertising and market research	No	No
23 Manufacture of other non-metallic products etc	No	No	74 Other professional activities etc	No	No
24 Manufacture of basic metals	No	No	75 Veterinary activities	No	No
25 Manufacture of fabricated products etc	No	No	77 Rental and leasing activities	Yes	Yes
26 Manufacture of computers etc	No	No	78 Employment activities	No	No
27 Manufacture of electrical equipment	No	No	79 Travel agency, tour operators etc	Yes	Yes
28 Manufacture of machinery etc	No	No	80 Security and investigation activities	No	No
29 Manufacture of motor vehicles etc	No	No	81 Services to buildings activities etc	No	No
30 Manufacture of other transport equipment	No	No	82 Office administrative activities	No	Yes
31 Manufacture of furniture	No	No	84 Public administration, etc	No	No
32 Other manufacturing	No	No	85 Education	No	Yes
33 Repair and installation of machinery etc	No	No	86 Human health activities	No	Yes
35 Electricity, gas, steam supply etc	No	No	87 Residential care activities	No	No
36 Water collection, treatment and supply	No	No	88 Social work activities etc	No	Yes
37 Sewerage	No	No	90 Creative activities etc	Yes	Yes
38 Waste collection, treatment activities	No	No	91 Libraries, archives, museums etc	No	Yes
39 Remediation activities and waste management	No	No	92 Gambling and betting activities	Yes	Yes
41 Construction of buildings	No	No	93 Sports activities etc	Yes	Yes
42 Civil engineering	No	No	94 Activities of membership organizations	No	Yes
43 Specialized construction activities	No	No	95 Repair of computers etc	No	No
45 Wholesale and retail trade etc	No	No	96 Other personal service activities	No	Yes
46 Wholesale trade, except motor vehicles etc	No	No	97 Activities of households etc	No	No
47 Retail trade, except motor vehicles etc	No	Yes	98 Undifferentiated goods and services	No	No
49 Land transport and transport via pipelines	Yes	No	99 Extraterritorial organizations etc	No	No

Notes: Treated sectors are those being tourism-related (grouped at the NACE Rev. 2, 2-digit level) according to the classification proposed by the International Recommendations for Tourism Statistics 2008, UN/UNWTO, http://unstats.un.org/unsd/publication/SeriesM/seriesm_83rev1e.pdf. Suspended sectors are based on the ELSTAT listing of sectors (grouped at the NACE Rev. 2, 2-digit level) under suspension as imposed by the Greek government in March 2020 (see https://www.statistics.gr/en/statistics/-/publication/SBR02/-)

### Daily job flows

We use daily data from ERGANI, an administrative database of the Ministry of Labor and Social Affairs (MoLSA), covering all salaried employees who contribute to the Social Security System. MoLSA publishes monthly reports on daily labor market flows using ERGANI data. They cover new hires (total, full-time, part-time, shift work) and separations (total, layoffs, quits, contract terminations) for the period 2018–2020. The total number of employees (aged $\ge$ 15 years old) is provided on an annual basis. The total daily stock of employees is constructed using the annual average number of employees in 2017 as the initial employment stock for January 1^st^, 2018, and the employment headcount at day $d$ was calculated as ${E}_{d}={E}_{d-1}+\left({H}_{d-1}-{S}_{d-1}\right)$, where $H$ denotes the daily number of new hires, and $S$ the daily number of new separations. Then, daily employment rates were constructed as the ratio of the daily employment stock over the monthly population using the official ELSTAT monthly working age (15–74 years old) raw population estimate. Using the daily employment stock ${E}_{d}$, we calculated the daily job hiring rate as ${h}_{d}={H}_{d}/\left[0.5\times \left({E}_{d}+{E}_{d-1}\right)\right]$. Similarly, the daily job separation rate was calculated as ${s}_{d}={S}_{d}/\left[0.5\times \left({E}_{d}+{E}_{d-1}\right)\right]$. Rates refer to total and particular types of new hires (full-time, part-time, and shift work) and separations (layoffs, quits, and contract terminations). We used these national daily time series for a first assessment of the short-term labor market responses to the pandemic onset and the mitigation measures imposed by the government. Under a DiD-style framework, we considered days between February 27 and June 30 in each year as the post period, and days from January 1 to February 26 as the period before. We defined 2020 as the treated year, exposed to the pandemic, and the years 2018–2019 serve as the control, pre-pandemic ones. We estimated models of the following form:

2 $${\Upsilon}_{dt}=\alpha +\beta {T}_{t}+\gamma {P}_{d}+\delta \left({{T}_{t}\times P}_{d}\right)+{w}_{d}+{m}_{d}+{\varepsilon }_{dt}$$

where, ${\Upsilon}_{dt}$ is the daily rate on day $d$ of year $t$, ${T}_{t}$ is a dummy indicating the treated year, ${P}_{d}$ is equal to 1 for the post period (February 27 – June 30) for every year and 0 otherwise, ${w}_{d}$ is a set of day-of-week fixed effects, ${m}_{d}$ is a set of monthly dummy indicators, and ${\varepsilon }_{dt}$ is the disturbance term. In Eq. (2), the parameter of interest, $\delta$, is associated with the interaction term $\left({{T}_{t}\times P}_{d}\right)$, indicating how daily labor market series changed in the early pandemic period relative to the same calendar period in the pre-pandemic years. For this approach to be valid, a common trends assumption needs to be satisfied, i.e. outcomes in the pre-exposure period to trend similarly in 2020 and in the previous years. We show, in Fig. 2, how this assumption is met for all types of outcomes.

Results are in Table 5 The full-time hiring rate in the pandemic year was 10.8 p.p. lower during the entire period after the pandemic onset, compared to the same period in the pre-pandemic years. This is also the case for part-time and for shiftwork hiring rates (9.3 p.p. and 3.0 p.p. lower, respectively). Overall, the total hiring rate in 2020, after the first verified COVID-19 case, was 23.1 p.p. lower compared to what was expected based on pre-pandemic trends, and almost 47% of this drop was due to the decrease in full-time hires. Regarding particular types of job separation, the layoff rate decreased by 5.1 p.p., the quit rate decreased by 2 p.p. and the contract termination rate decreased by 6.9 p.p. These changes led to a reduction in the overall separation rate by 14 p.p. The overall employment rate was 7.8 p.p. lower during the early pandemic period compared to what was expected in this period if the 2018–2019 trends had prevailed in 2020. These results provide a first indication that the observed employment decline was shaped entirely by the collapse in job hiring rather than increases in job separations. Results are identical when Eq. (2) also includes an Easter holiday binary indicator.

Figure 8 plots a more detailed picture for all outcomes reported in Table 5. More specifically, using Eq. (2), we interacted the treated year indicator, ${T}_{t}$, with weekly indicators, restricting the sample to the period January 1^st^, 2019 – June 30^th^. Hence, we compared the difference in outcomes for the same week between 2020 and 2019. First, we notice that before the exposure period, outcomes trended similarly for the first few weeks of each year. Therefore, the estimates presented in Table 1 do not simply pick up some pre-existing differential trend. Employment rate responded immediately to the pandemic onset, i.e. the week when the first COVID-19 case was verified. Since then, as the government imposed a set of policies including workplace restrictions (March 13, 2020) and layoff restrictions (a few days later), the employment rate continued to decrease and it gradually dropped by 12 p.p. relative to 2019, around the second week of May. By the end of May, the employment rate levelled off and it started rising as restrictions were being relaxed. Comparing the actual employment to the counterfactual employment levels, suggests an estimated cumulative job deficit of 265,000 by the end of June 2020. Interestingly, in the week when the first COVID-19 case was reported, we observe an immediate negative response in the job hiring rate, but not in the job separation rate. The reduction in job hiring peaked during the second and third weeks of April 2020, which are the weeks when new hires increased most during the pre-pandemic year as the tourism sector geared up. New hires in 2020 continued to be lower compared to the pre-pandemic scenario until the end of May when the restrictions were gradually lifted. Moreover, job separations were reduced a few weeks after the onset of the pandemic and its growth rate remained negative throughout the first pandemic wave. A weak, slightly positive trend in job separations during the first non-pandemic months of 2020 reversed right after the introduction of the government policies regarding job retention in suspended sectors.

### Daily vacancies

We also use daily vacancy data from a popular online portal (www.jobfind.gr) to complement the evidence on how the Greek labor market responded before and after the pandemic onset. Using web crawling techniques, we collected 5,471 unique job postings from all over the country (multiple postings from the same firm and with the same text content were removed). Figure 9. plots the daily vacancy rate between January 1st, 2020, and May 30th, 2020. In this way, we can obtain a picture of how it evolved before and after the pandemic onset. Our limitations here are that job postings data were not available before 2020, and that there is no information on the sector of economic activity for each firm that posted a job opening. However, the evidence is consistent with the sharp decline in new hires documented in Sect. 2. The sharp decrease in vacancies coincides with the implementation of workplace restrictions in mid-March. In addition, there was a slight increase in job postings in May as restrictions were lifted, but new postings remained far below their pre-pandemic level.

A concern here could relate to the representativeness of the information gathered online, especially when compared with official vacancy data. This sort of comparison is challenging due to three sources of bias, i.e. aggregate stock bias, online representativeness bias, and job-related bias (Turrell et al. 2021), and it is beyond the scope of this paper. Additionally, official vacancy data are provided by the Eurostat only on a quarterly frequency. However, they also point towards a reduced vacancy rate from 0.4% in 2020Q1 to 0.3% in 2020Q2. Also, they allow for a comparison of the occupied jobs and vacancy shares between 2020Q1 and 2020Q2 (see Appendix Table 6). The most pronounced vacancy share decrease, relative to one year back, comes from the accommodation and food services sector, by 25 p.p. and 33 p.p. in 2020Q1 and 2020Q2, respectively. On the other hand, the share of occupied jobs in this sector remained unchanged over the same period. This further strengthens our argument about the short-run labor market impact of the pandemic coming mainly through reduced hiring, especially in tourism-related services.

## Footnotes

1 The timing of the pandemic onset and the subsequently imposed mitigation measures interrupted those patterns, shutting down hiring during the months when it normally would be the strongest within the year. Moreover, behavioral adjustments and mobility restrictions were also observed in countries on which the country’s total demand for tourism-related goods and services normally depends on, e.g. accommodation and food services.

2 Since 2017, after several years of contraction, the average annual GDP growth rate was 1.5%. At the same time, unemployment was declining, although at the end of 2019, it was the highest among the OECD countries, i.e. 16.5%.

3 Regarding the process of labor reallocation, this was of paramount importance because any employment losses would not be due to increased layoffs. In other words, the adopted job retention policies in Greece maintained job matches and prevented further rises in unemployment. In addition, due to the job retention schemes the number of displaced workers who had to search for new jobs did not increase. On the other hand, the pre-pandemic temporary hiring patterns was severely affected due to revised expectations about significantly lower demand, especially for employers in sectors that were mostly affected, e.g. accommodation, restaurants etc., where job requirements do not allow for remote or other flexible ways of working.

4 On the aggregate level, we confirm the above using administrative daily data on employment, hires, and separations, and online daily vacancies data. For example, employment declined during the early days of the pandemic compared to what we would have expected given trends observed in previous years, and vacancies were lower in regions heavily exposed to hospitality services.

5 Amuedo-Dorantes et al. (2021) discuss the importance of implementing social-distancing measures early, given the absence of effective vaccines during the pandemic onset.

6 For the impact of government-imposed social distancing mandates on human mobility worldwide, see Mendolia et al. (2021).

7 Early projections from national and international institutions estimated that GDP would shrink between 5.7% and 10% in 2020, primarily because of a decline in exports, and especially tourism and shipping (KEPE 2020).

8 More formally, let *Y* to denote some labor market outcome in a given month. Following Lemieux et al. (2020), the year-to-year comparison is calculated as [(Y_C,2020_ – Y_B,2020_)/(Y_B,2020_)] – [(Y_C,2019_ – Y_B,2019_)/(Y_B,2019_)], where *C* is the current month, and *B* is the base month (February) of the respective year. The within-year difference is the percentage difference in *Y*, and the double differences represent the changes in 2020 over and above the base month (*B*) – current month (*C*) changes that would be expected to occur as proxied by the 2019 realizations. According to these calculations, in May 2020, employment and labor force participation were lower by 5%-6% and 2%-3%, respectively, while unemployment was higher by 12.9% compared to a pre-pandemic year. It should be noted that these estimates refer to individuals aged 15–74 years old, including public sector employees and self-employed individuals.

9 About 800,000 firms that were affected in terms of a decline in their turnover or had ceased operation by state order were eligible for these and other benefits described below, on the condition of no layoffs.

10 In addition, for these workers, the government covered all social insurance contributions, and all tax payments were suspended for a period of 4 months. The same measure was applied to freelancers, self-employed, and individual business owners with up to 20 employees. Firms in suspended sectors and affected employees were asked to pay just 60% of their rents for March to May.

11 A one-off stipend of €600 in the form of a special training program was provided to specific professionals (economists/accountants, engineers, lawyers, doctors, teachers, and researchers) in April. These occupations became eligible for the €800 financial support as of May. The budget allocation for the stipends for employees, freelancers/self-employed/individual businesses, and professionals was €2.36 billion, with an additional €1.36 billion for the social insurance payments.

12 Monthly Public Employment Service (OAED) data on the number of unemployment benefit recipients indicate the same story. More specifically, they reflect the seasonality of the Greek labor market, with the total number of beneficiaries falling in the spring, with few new claims. In 2020, after slightly increasing in February and March, new claims tripled in April before reverting to a level more comparable to previous years in May (and even below in June). Nevertheless, the adverse pandemic impact on unemployment stock is further confirmed by the number of paid beneficiaries during the first half of 2020 which was higher than in previous years.

13 The budget allocation for these measures related to unemployment benefits was nearly €300 million.

14 Figure 10 in the Appendix shows the monthly evolution of arrivals in hotels and similar establishments by region (2017M1-2020M6).

15 ELSTAT does not provide these data at the regional level.

16 For all outcomes considered here, we have also estimated Eq. (1) separately for suspended and non-suspended sectors. The results show that the impact is practically identical in both sub-samples, for every outcome considered. Therefore, the results we observe, are due to a sector being treated, rather than being suspended. Results are available upon request.
